# Comparative analysis of two independent *Myh6-Cre* transgenic mouse lines

**DOI:** 10.1016/j.jmccpl.2024.100081

**Published:** 2024-07-03

**Authors:** Amanda Davenport, Chase W. Kessinger, Ryan D. Pfeiffer, Nikita Shah, Richard Xu, E. Dale Abel, Nathan R. Tucker, Zhiqiang Lin

**Affiliations:** a Department of Biomedical Research and Translational Medicine, Masonic Medical Research Institute, 2150 Bleecker Street, Utica, NY 13501, United States of America; b College of Arts and Sciences, SUNY Polytechnic Institute, Utica, NY 13502, United States of America; c Department of Medicine David Geffen School of Medicine and UCLA Health, United States of America

**Keywords:** Cre-loxP system, Myh6-Cre, tdTomato reporter, Transgenic mouse line, Genomic position effect

## Abstract

We have previously shown that the *Myh6* promoter drives Cre expression in a subset of male germ line cells in three independent *Myh6-Cre* mouse lines, including two transgenic lines and one knock-in allele. In this study, we further compared the tissue-specificity of the two *Myh6-Cre* transgenic mouse lines, *MDS Myh6-Cre and AUTR Myh6-Cre,* through examining the expression of tdTomato (tdTom) red fluorescence protein in multiple internal organs, including the heart, brain, liver, lung, pancreas and brown adipose tissue. Our results show that *MDS Myh6-Cre* mainly activates tdTom reporter in the heart, whereas *AUTR Myh6-Cre* activates tdTom expression significantly in the heart, and in the cells of liver, pancreas and brain. In the heart, similar to *MDS Myh6-Cre***,***AUTR Myh6-Cre* activates tdTom in most cardiomyocytes. In the other organs, *AUTR Myh6-Cre* not only mosaically activates tdTom in some parenchymal cells, such as hepatocytes in the liver and neurons in the brain, but also turns on tdTom in some interstitial cells of unknown identity.

## Highlights

• MDS Myh6-Cre and AUTR Myh6-Cre are two independent mouse lines used for heart-specific studies
• MDS Myh6-Cre and AUTR Myh6-Cre mouse lines both have Cre activity in the heart and testis
• AUTR Myh6-Cre has ectopic Cre activity in brain, liver and pancreas
• Whether AUTR Myh6-Cre causes non-heart-specific phenotype depends on the nature of the flox alleles.

## Graphical abstract

**Figure:**
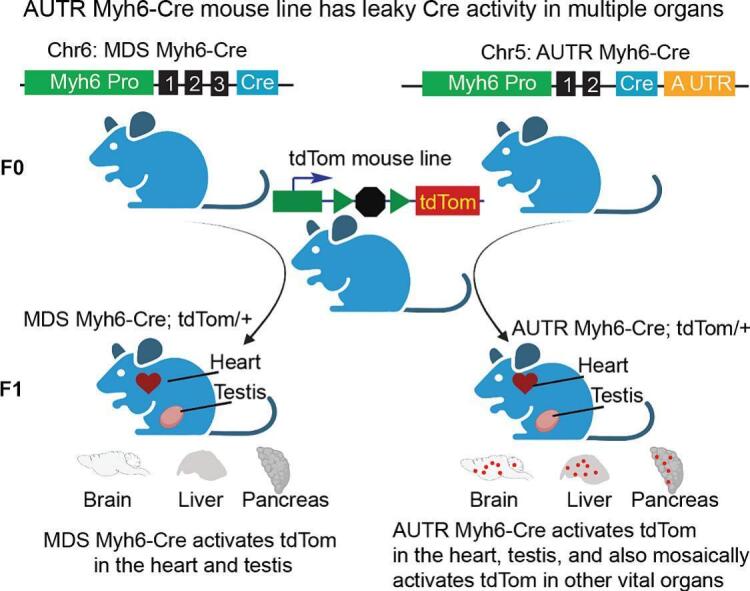

## 1 Introduction

The Cre-LoxP system has been widely used to genetically manipulate gene expression to perform gain-of-function and loss-of-function studies, so that the candidate genes can be studied in various tissues at different developmental stages [1]. In this system, the expression of Cre recombinase is driven by tissue-specific promoters, and the Cre recognizable loxP DNA sequences are engineered into the genome to flank a crucial DNA fragment that controls the expression of a target gene [2]. The tissue-specific Cre driver mouse lines are sometimes transgenic, with the promoter+Cre DNA sequence randomly inserted into the genome. During generation of the Cre driver mouse line, several independent transgenic lines can be produced, of which the transgenic DNA fragments are localized in different genomic loci. By characterizing the expression profile of the Cre gene, one can screen out the transgenic mouse line that reliably expresses Cre in the desired tissue.

Although Cre expression is under tight control of the tissue-specific promoter in most transgenic Cre lines, some Cre driver lines unexpectedly express Cre in undesired tissues [3], a phenomenon called leaky expression. If not noticed, the aberrant expressed Cre can potentially confound analysis of the tissue-specific gene function study and cause enigmatic phenotypes [4]. The gold standard to validate the tissue-specificity of a Cre driver mouse line is to cross it with a reporter mouse allele, which does not express the reporter gene unless Cre recombinase is expressed. Rosa26^tdTom^ (tdTom), also known as Ai14, is a Cre reporter strain containing a loxP-flanked STOP cassette in front of the tdTomato (tdTom, a red fluorescence protein) gene. This loxP-flanked STOP cassette prevents tdTom expression in the absence of Cre recombinase; once Cre is provided, the Cre recombinase will recognize the loxP sequences and trigger DNA recombination, leading to the deletion of the STOP cassette and activation of the tdTom gene [5]. When a Cre driver strain is crossed with the tdTom reporter mouse line to generate Cre-driver+tdTom mice, the Cre-expressing cells of these Cre-driver+tdTom mice will produce easily detectible tdTom fluorescence protein, thereby accurately mirroring the spatial distribution of the cells that express Cre and the cells that are derived from Cre-positive progenitors.

In the past two decades, a few cardiomyocyte (CM) specific Cre driver lines have been generated to study gene function in CMs during heart development and disease progression. One of the most widely used promoters that drives Cre expression in CMs is derived from the myosin heavy chain 6 and 7 (*Myh6-Myh7*) gene locus [6]. Five *Myh6* promoter-based driver lines have been generated, including three constitutively active Cre lines (*Myh6-Cre*) [[7], [8], [9]] and two tamoxifen inducible strains (*Myh6-MerCreMer*) [10,11]. Among the three *Myh6-Cre* lines, two are transgenic [7,8] and one is a recently developed knock-in allele [9].

The two transgenic *Myh6-Cre* mouse lines share the same *Myh6* promoter region and differ due to the presence of a human β-actin untranslated region (UTR) in the 3 prime side of the Cre gene. To discriminate these two transgenic *Myh6-Cre* lines, we named the line that contains human β-actin UTR as *AUTR Myh6-Cre* [8]. The other transgenic *Myh6-Cre* line does not contain β-actin UTR and was generated by Michael D. Schneider's group, and therefore was named as *MDS Myh6-Cre* for this study [7].

Our previous work has shown that male *Myh6-Cre* mice express Cre in the germ line cells. When the *Myh6-Cre* mice are crossed with tdTom reporter, the first generation (F1) male *Myh6-Cre+/−;tdTom* mice produce some tdTom-expressing sperms, of which the loxP-flanked STOP cassette in front of the tdTom gene has already been permanently removed by Cre. In the second generation (F2), when male *Myh6-Cre+/−;tdTom* are crossed with any genotype female mice, the tdTom-expressing sperms will produce offspring that universally express tdTom [12]. Due to the strong expression of tdTom red fluorescence protein in the skin, the whole body tdTom-labeled mice can be easily separated from the normal *Myh6-Cre*+/−;tdTom mice that selectively express tdTom gene in the Cre positive organs [12].

During examination of tdTom reporter gene expression patterns in the normal *MDS Myh6-Cre*;tdTom and *AUTR Myh6-Cre*;tdTom mice, we found that these mice displayed different tdTom expression profiles, which was not due to germline activation of the tdTom reporter gene. Our results show that *MDS* Myh6-Cre specifically activated tdTom in the heart. Although *AUTR Myh6-Cre* efficiently activated tdTom in the heart, it also displayed ectopic Cre activity in the other organs, especially in the brain and liver. Using Targeted Locus Amplification (TLA) technology, we identified the integration loci of these two *Myh6-Cre* mouse lines, and our results suggest genomic position effect may impair the tissue-specificity of *the AUTR Myh6-Cre* mouse line.

## 2 Materials and methods

### 2.1 Supporting information provides extended protocols

#### 2.1.1 Mice

All animal procedures were approved by the Institutional Animal Care and Use Committee of Masonic Medical Research Institute. Rosa26^tdTom^ (tdTom) [5], *MDS Myh6-Cre* [7] and *Yap*^*fl/fl*^ [13] mouse lines were all purchased from Jackson Laboratory. *AUTR Myh6-Cre* [8] was a gift from Dr. E. Dale Abel.

#### 2.1.2 Myh6-Cre genotyping and DNA recombination detection

With mouse tail DNA as template, PCR-based genotyping was performed with the following two primers Pr-I: ATGACAGACAGATCCCTCCTATCTCC; Pr-II: CCCTGAACATGTCCATCAGG**.** For tdTom recombination detection, these primers were used: Pr-1, CTCCGAGGACAACAACATGG; Pr-2, CGTCCATGCCGTACAGGAAC.

Pr-3, 5’-TTCTGTGGCTGCGTGAAAG-3′; Pr-4, GCCGTCCTCGAAGTTCATCA**.**

The following PCR program was used for both genotyping and DNA recombination detection:

95 °C (5 min); 95 °C (30 s); 58 °C (30 s), 72 °C (1 min); 34 cycles of [95 °C (30 s); 58 °C (30 s), 72 °C (1 min)]; 72 °C (5 min); 15 °C (1 min).

#### 2.1.3 Whole-body and ex vivo fluorescence imaging

Fluorescence ex vivo organ imaging was performed on an IVIS Spectrum imaging system (Perkin Elmer, Waltham, MA). Image acquisition utilized Living Image software (4.5.5, Perkin Elmer) with fluorescence from tdTomato reported as radiant efficiency. For ex vivo organ imaging, mice were saline perfused, and tissues of interest were resected and placed on a black plastic substrate for imaging. All Images were collected with a field of view of 13.2cm^2^ using 570/620 nm fluorescence excitation/emission filters with an exposure time of 0.75–2 s.

#### 2.1.4 Histology

Organs were fixed in 4 % PFA, cryoprotected with 30 % sucrose and embedded in OCT. Imaging was performed on a Keyence BZ-X800 microscopy system.

#### 2.1.5 Brain section NeuN immunofluosrecence staining

Brain cryosections of 50 μm thickness were permeabilized with 1 % PBST for half an hour, blocked with 5 % normal donkey serum for 2 h at room temperature, and then incubated with NeuN antibody (Proteintech, 26,975–1-AP, 1:200) at 4 °C overnight. On the second day, after three rounds 0.1 % PBST wash, donkey anti-rabbit Alexa fluor 488 secondary antibody was applied to label cells expressing NeuN. Imaging was performed on a Keyence BZ-X800 microscope system or a Nikon AXR Confocal system.

#### 2.1.6 Targeted locus amplification (TLA)

TLA was performed on murine liver nuclear extracts as described [14], with minor modifications to facilitate specific amplification of the Myh6-Cre transgenic construct. Detailed methods for TLA library production and analysis are contained within the supplemental information. TLA Sequencing data were deposited into NIH's Sequence Read Archive (SRA), with an SRA bioproject ID: PRJNA1123655.

## 3 Results

### 3.1 Comparison of the tissue-specificity of *MDS Myh6-Cre* and *AUTR Myh6-Cre transgenic* strains

YAP is one of the essential terminal effectors of the Hippo-YAP pathway [15], and knocking out YAP with the *MDS Myh6-Cre* causes heart failure and premature death (a median lifespan between 10 and 11 weeks) [16]. During studying YAP function in the postnatal heart, we crossed *AUTR Myh6-Cre* and *MDS Myh6-Cre* with mice harboring the *Yap* flox allele [13] to generate *AUTR Myh6-Cre*;*Yap*^*fl/fl*^ (*AUTR Yap*^*cKO*^) and *MDS Myh6-Cre*;*Yap*^*fl/fl*^ (*MDS Yap*^*cKO*^) mice, respectively (Suppl. Fig. 1A). In the first generation (F1), male *MDS Myh6-Cre* mice were crossed with *Yap*^*fl/fl*^, to get *MDS Myh6-Cre*;*Yap*^*fl/+*^ (*MDS Yap*^*cHet*^). In the second generation (F2), *MDS Yap*^*cHet*^ mice were crossed with *Yap*^*fl/fl*^ to get *MDS Yap*^*cKO*^*.* In the third generation (F3), male *MDS Yap*^*cKO*^ mice were bred with female *Yap*^*fl/fl*^ to expand the *MDS Yap*^*cKO*^ colony. During all these breedings, the observed frequency of *MDS Yap*^*cHet*^ and *MDS Yap*^*cKO*^ followed a mendelian ratio*.* For example, we quantified the *MDS Yap*^*cKO*^ genotype frequency in eight litters of F3 weaned pups, of which 26 were *Yap*^*fl/fl*^ and 24 were *MDS Yap*^*cKO*^ (Suppl. Fig. 1B).

We used the same breeding strategy to obtain *AUTR Yap*^*cKO*^ mice. In the F2 pups, we had difficulty in obtaining *AUTR Yap*^*cKO*^ mice. After several rounds of breeding, we managed to obtain two male *AUTR Yap*^*cKO*^ mice that survived to adulthood. Nevertheless, when these male *AUTR Yap*^*cKO*^ were crossed with female *Yap*^*fl/fl*^ mice to generate F3 offspring, only two were *AUTR Yap*^*cKO*^ in a total number of 62 weaned pups (Suppl. Fig. 1B). These results suggest that the *AUTR Yap*^*cKO*^ mice either die during embryo stage or cannot prevail to weaning stage.

**Fig. 1:**
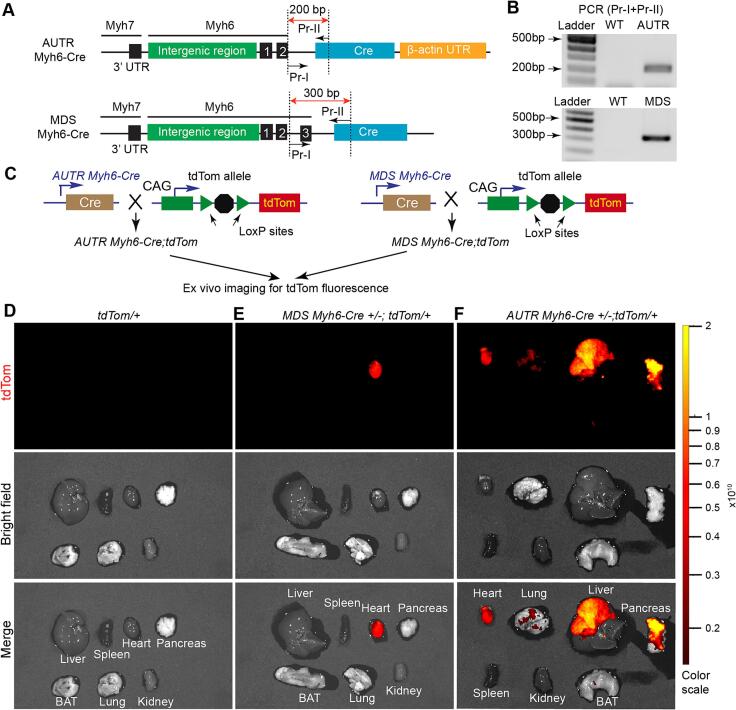
***AUTR Myh6-Cre activates tdTom reporter in multiple internal organs.* A**. Schematic representation of the targeting constructs used to generate Myh6-Cre transgenic mouse lines. Numbered black bars indicate *Myh6* gene exons. Dotted lines outline the PCR amplicons used for genotyping. **B**. PCR-based genotyping. Mouse Tail DNA was used as template. **C.** Breeding scheme that leads to the production of *AUTR Myh6-Cre*;*tdTom and MDS Myh6-Cre; tdTom.***D—F**. Ex vivo fluorescent imaging of mouse organs. 2-month old mice were included in this study. Fluorescent imaging was performed with Perkin Elmer IVIS Spectrum system. Colour range indicates radiant efficiency ([p/s/cm^2^/sr]/[μW/cm^2^]).

One possibility for the reduced lifespan in *AUTR Yap*^*cKO*^ could be ectopic Cre expression. To test this hypothesis, we systematically compared the tissue-specificity of *AUTR Myh6-Cre* and *MDS Myh6-Cre*. Previously, the heart-specificity of *MDS Myh6-Cre* allele was defined by crossing *MDS Myh6-Cre* to the CAG-CATZ reporter mouse line, which carries a Cre-inducible LacZ reporter gene [7]; however, the heart-specificity of *AUTR Myh6-Cre* has not been defined with genetic reporter mouse lines. Compared with *AUTR Myh6-Cre*, *MDS Myh6-Cre* construct has an additional *Myh6* exon 3 splice acceptor sequence located at 180 bp upstream of the Cre gene [7] (Fig. 1A). We designed two primers (Pr—I and Pr-II) that annealed to the *Myh6* exon 2 downstream sequence and the 5 prime side of the Cre sequence, respectively (Fig. 1A), so that a PCR test can be used to discriminate these two transgenic mouse lines (Fig. 1B).

To compare the tissue specificity of these two *Myh6-Cre* lines, we crossed them to the Rosa26^tdTom^ (tdTom) reporter mouse line [5], which does not express tdTom until the Cre recombinase is provided. After genotyping, *AUTR Myh6-Cre;tdTom* and *MDS Myh6-Cre;tdTom* mice (Fig. 1C) were subjected for fluorescence reporter analysis. At 2–3 months after birth, we collected the internal organs and brown adipose tissue (BAT) for ex vivo fluorescence imaging. As expected, in the absence of Cre, no tdTom fluorescence signal was detected in the tdTom reporter mouse organs (Fig. 1D). In all the examined organs of the *MDS Myh6-Cre;tdTom* mice, only the heart showed strong tdTom fluorescence signals (Fig. 1E). In contrast to the *MDS Myh6-Cre;tdTom* mice, the *AUTR Myh6-Cre;tdTom* mice displayed easily discernible tdTom fluorescence signals in multiple organs, including the heart, liver and pancreas (Fig. 1F).

### 3.2 *AUTR Myh6-Cre* induces DNA recombination in different tissues

**Fig. 2:**
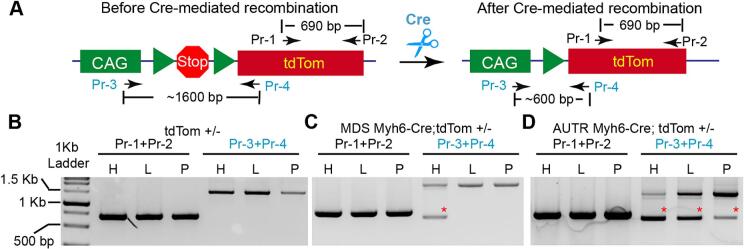
***AUTR Myh6-Cre* depletes the stop cassette of the tdTom transgenic construct in multiple organs. A**. Depiction of the PCR strategy for detecting Stop cassette depletion. Pr-1 and Pr-2 primer pair was used to detect the tdTom DNA sequence. Pr-3 and Pr-4 primer pair was used to examine the occurrence of Cre-mediated Stop cassette depletion. **B**—**D**. PCR results. Genomic DNA from different organs was used as PCR template. H, heart; L, liver, P, Pancreas. Red stars indicate the PCR amplicons derived from the recombined tdTom reporter allele. (For interpretation of the references to colour in this figure legend, the reader is referred to the web version of this article.)

To corroborate our observation on the molecular level, we designed two sets of primers to examine the recombination of the transgenic tdTom DNA construct. The first pair of primers (Pr-1 and Pr-2) annealing to the tdTom gene sequence was used as positive control, and their PCR products were around 690 bp. For the second pair of primers, Pr-3 annealed to the CAG promoter's 3 prime region, and Pr-4 annealed to the 5 prime side of the tdTom gene (Fig. 2A). Before introducing Cre recombinase, the Pr-3 + Pr-4 PCR amplicon was around 1600 bp (Fig. 2A). After Cre-mediated removal of the floxed stop cassette, the PCR product of Pr-3 + Pr-4 changed from 1600 bp to 600 bp (Fig. 2B). As expected, Pr-1 + Pr-2 PCR products were all the same among different tissues of the tdTom, *AUTR Myh6-Cre;tdTom* or *MDS Myh6-Cre;tdTom* mice (Fig. 2B, C, and D). No DNA recombination event was detected in the tdTom mouse tissues (Fig. 2C). In the *MDS Myh6-Cre;tdTom* mice, tdTom DNA recombination occurred in the heart but not in the liver and pancreas (Fig. 2C). Consistent with the ex vivo imaging results, tdTom DNA recombination was detected in the heart, liver and pancreas of the *AUTR Myh6-Cre;tdTom* mice (Fig. 2D).

### 3.3 *The AUTR Myh6-Cre* strain expresses Cre in CMs and in the interstitial cells of different organs

**Fig. 3:**
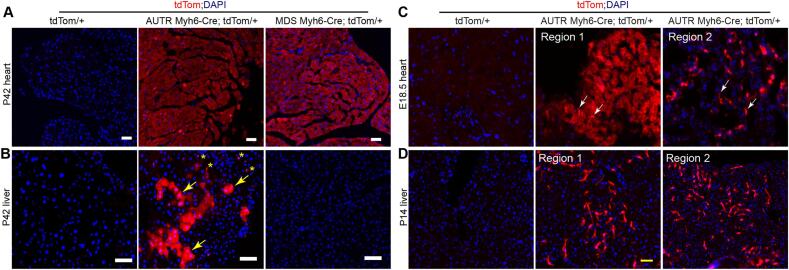
***AUTR Myh6-Cre* activates tdTom in cardiomyocytes (CMs) and other cell types. A-B.** tdTom fluorescence images of the adult heart (A) and liver (B) sections. P42, postnatal day 42. B, yellow arrows indicate hepatocytes; yellow stars indicate non-hepatocytes. **C.** tdTom *and AUTR Myh6-Cre*;*tdTom* heart sections at embyronic stage 18.5 (E18.5). White arrows indicate non-CMs. Two myocardial regions from the same heart are presented. **D**. tdTom *and AUTR Myh6-Cre*;*tdTom* liver sections at P14. Two regions from the same liver section are presented. A, B, C and D, scale bar = 50 μm. (For interpretation of the references to colour in this figure legend, the reader is referred to the web version of this article.)

The ex vivo imaging and the tdTom transgenic DNA recombination results both suggest that the *AUTR Myh6-Cre* aberrantly expresses Cre in multiple organs. We then attempted to define the cell types that ectopically express Cre in the *AUTR Myh6-Cre* mice. With the *MDS Myh6-Cre;tdTom and AUTR Myh6-Cre;tdTom* mice, we first examined the possible parenchymal cells that express Cre in the adult heart (Fig. 3A) and liver (Fig. 3B)*.* In the myocardia of the adult *MDS Myh6-Cre;tdTom and AUTR Myh6-Cre;tdTom* mice, the CMs were all tdTom positive and had a similar tdTom fluorescence intensity (Fig. 3A). In line with the ex vivo fluorescence imaging data, the *MDS Myh6-Cre;tdTom* liver sections did not contain tdTom positive cells (Fig. 3B), whereas the *AUTR Myh6-Cre;tdTom* liver sections carried many tdTom fluorescence-labeled polygonal shaped hepatocytes and irregular shape interstitial cells (Fig. 3B). Moreover, in the *AUTR Myh6-Cre;tdTom* mice, we found tdTom+ interstitial cells not only in the adult liver but also in the fetal heart, in which a subset of the fetal CMs and some irregular shaped interstitial cells were labeled with tdTom (Fig. 3C). We then expanded our observation to other tissues collected from two-week-old *AUTR Myh6-Cre;tdTom* mice, which should have well-formed organs and terminally differentiated parenchymal cells. Interestingly, although both tdTom+ hepatocytes and tdTom+ interstitial cells existed in the adult liver (Fig. 3B), only tdTom+ interstitial cells were detected in the livers of the two-week-old *AUTR Myh6-Cre;tdTom* mice (Fig. 3D).

When performing ex vivo imaging with the *AUTR Myh6-Cre;tdTom* tissues, we did not detect strong tdTom fluorescence in the lung, brown adipose tissue, and spleen; however, the tdTom+ cells were detected in all of these tissues by miscroscopic analysis of the tissue sections (Suppl. Fig. 2A and B). Besides the heart and liver, the pancreas was the other organ displaying strong ex vivo tdTom fluorescence. Correspondingly, the adult *AUTR Myh6-Cre;tdTom* pancreas contained many polygonal shape tdTom+ cells (Suppl. Fig. 2B), which appeared more like acinar cells than clustered Langerhans islet cells. Together, these data suggest that the *AUTR Myh6-Cre* transgenic mouse line ectopically expresses Cre in hepatocytes and in undefined cell types of multiple organs.

### 3.4 The *AUTR Myh6-Cre* strain has Cre recombinase activity in brain

**Fig. 4:**
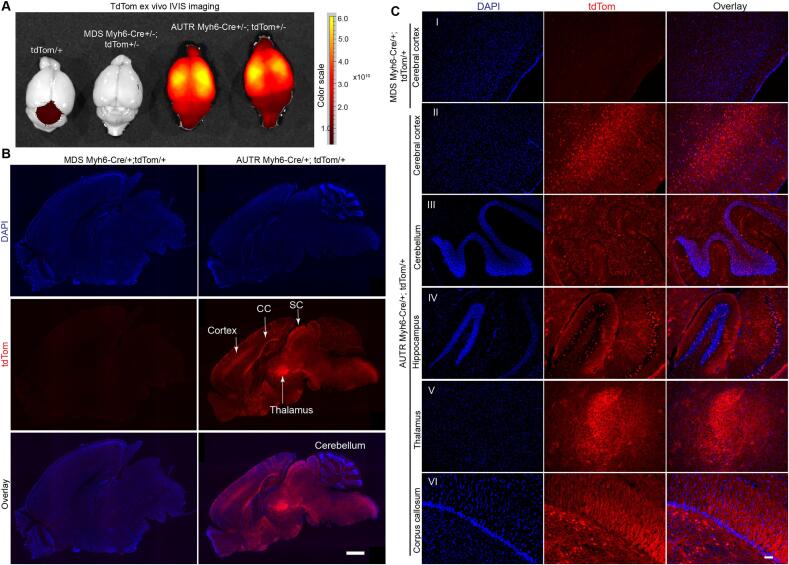
***AUTR Myh6-Cre activates tdTom reporter in the brain.* A.** Ex vivo fluorescent imaging of brain. 2-month old mice were included in this study. Colour range indicates radiant efficiency ([p/s/cm^2^/sr]/[μW/cm^2^]). **B—C**. tdTom fluorescence images of the sagital brain sections. B, tdTom signal distribution in the whole brain. CC, corpus callosum; SC, superior colliculus. Scale bar = 1000 μm. C, magnified images of tdTom positive cells in different brain regions. Scale bar = 50 μm.

In addition to the heart, lung, brown adipose tissue and the abdominal organs, we also examined the brain for tdTom signals. Surprisingly, the *AUTR Myh6-Cre;tdTom* mouse brain exhibited gross tdTom fluorescence, whereas the brain from tdTom and *MDS Myh6-Cre;tdTom* mice only showed some non-specific background autofluorescence (Fig. 4A). We sectioned the brains to examine the distribution of tdTom+ cells in detail. Brain sagittal sections showed that the *MDS Myh6-Cre;tdTom* brain had low background non-specific autofluorescence. Nevertheless, the *AUTR Myh6-Cre;tdTom* brain was highly fluorescent, with tdTom+ cells distributed in the cerebral cortex, corpus callosum, thalamus and the superior colliculus of midbrain (Fig. 4B). To better characterize the tdTom+ cells distribution patterns in different brain regions, we acquired magnified fluorescence images of the cerebral cortex, cerebellum, hippocampus, thalamus and corpus callosum. *MDS Myh6-Cre;tdTom* cerebral cortex was used as negative control for setting the fluorescence imaging exposure time (Fig. 4C-I). In the cerebral cortex, the tdTom+ cells were enriched in the anterior cingulate cortex [17] region and sparsely distributed elsewhere (Fig. 4B and C-II); in cerebellum, the inner granular layers were enriched with tdTom+ cells (Fig. 4C-III); in the hippocampus formation, tdTom+ cells were dispersedly distributed in the granular layer (Fig. 4C-IV). In the thalamus, strong tdTom fluorescence was detected (Fig. 4C-V), and the fluorescence signals did not overlap with DAPI (nuclei marker). The corpus callosum also showed strong tdTom fluorescence signals, which did not overlap with DAPI (Fig. 4C-VI). These data suggest that *AUTR Myh6-Cre* ectopically expresses Cre in the brain cells, causing the activation of tdTom reporter in different brain regions.

### 3.5 *AUTR Myh6-Cre* activates tdTom expression in a sub-population of neurons

In mammalian brain, the corpus callosum is the major neuron axonal tract that connects the brain hemispheres [18], and it was strongly labeled with tdTom in the *AUTR Myh6-Cre;tdTom* brain. Of note, the *AUTR Myh6-Cre* mosaically activated tdTom in the brain. These observations prompted us to hypothesize that *AUTR Myh6-Cre* drives Cre expression in a subset of neuronal cells. To test this hypothesis and corroborate our observations, we stained the *AUTR Myh6-Cre;tdTom* brain with NeuN antibody, a molecular marker that labels the nuclei of mature neurons [19], and acquired high resolution confocal images from the olfactory bulb, cerebral cortex, hippocampus and cerebellum cortex.

**Fig. 5:**
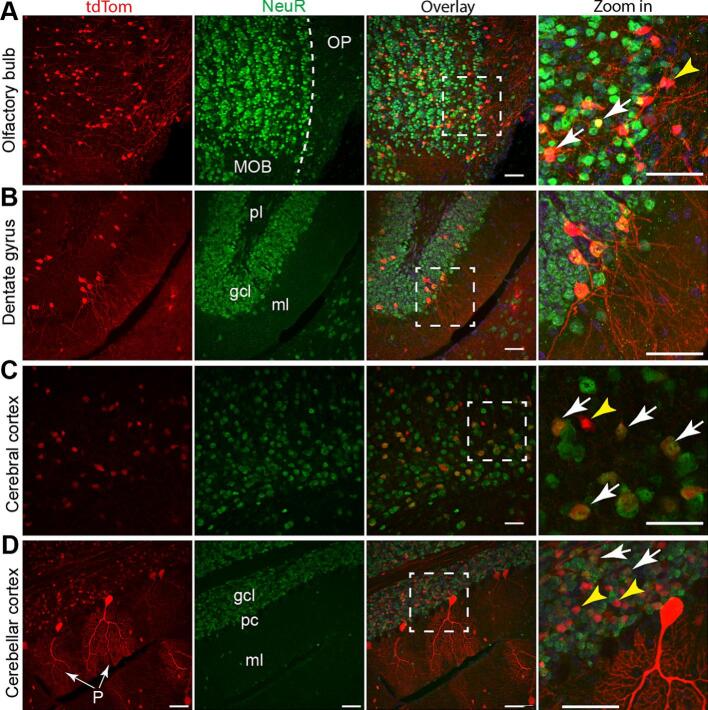
**AUTR *Myh6-Cre* activates tdTom reporter in both neurons and non-neuron cells.** Confocal images of olfactory bulb (**A**), hippocampus dentate gyrus (**B**), isocortex (**C**) and cerebellar cortex (**D**). NeuN antibody was used to label the cell bodies of mature neurons. White arrows indicate tdTom positive mature neurons, and yellow arrowheads indicate non-neuron cells. A, MOB: main olfactory bulb; OP; olfactory peduncle. B, ml: molecular layer; gcl: granule cell layer; pl: polymorphic layer (pl). In panel D, P indicates Purkinje cells. Scale bar = 50 μm. (For interpretation of the references to colour in this figure legend, the reader is referred to the web version of this article.)

In the olfactory bulb, most of the tdTom+ cells were localized in the main olfactory bulb (MOB), with a small portion of them positive for NeuN and the rest lacking NeuN signals (Fig. 5A). The Olfactory Peduncle (OP) is a stalk tissue connecting the olfactory bulb with the basal forebrain [20], tdTom+ neuron fibers were present in the OP (Fig. 5A). As one part of the hippocampus formation, the dentate gyrus contains three layers: molecular layer (ml) composed of neuron fibers, granule cell layer (gcl), and a polymorphic (pl) cell layer [21]. In the dentate gyrus, a small number of tdTom-labeled mature dentate granule cells were sparsely distributed in the granule cell layer (Fig. 5B). In the cingulate cortex of the cerebrum, most of the tdTom+ cells were NeuN positive; however, a small population of Tom+;NeuN - cells also exist (Fig. 5C). We quantified the percentage of Tom+;NeuN+ cells in the anterior cingulate cortex and found that 22.61 ± 5.2 % neurons were labeled by tdTom (Suppl. Fig. 3). In the cerebellum cortex, most of the tdTom+ cells were populated in the granule cell layer and some tdTom+ cells were identified as Purkinje cells due to their highly branched dendritic tree structure; in the granule cell layer, tdTom+;NeuN+ and tdTom+;NeuN cells were both detected (Fig. 5D). Together, these data suggest that *AUTR Myh6-Cre* activates tdTom expression in a sub-population of brain cells, which include both mature neurons and non-neuron cells. (See Fig. 5.)

### 3.6 Identification of AUTR Myh6-Cre genomic integration locus

Cardiac specific cis-regulatory sequences are almost identical between *AUTR Myh6-Cre* and *MDS Myh6-Cre;* however, these two Cre-driver lines demonstrated different Cre expression specificity. We reasoned that genomic locus effects might underlie the different expression patterns in these two transgenic alleles: for the *AUTR Myh6-Cre* mouse line, the *Cre* gene expression could be regulated by both *Myh6* promoter and other unknown cis-regulatory elements around the *AUTR Myh6-Cre* insertion locus; whereas for the *MDS Myh6-Cre* mouse line, *Cre* gene expression is tightly controlled by *Myh6* promoter and no functional cis-regulatory elements are present around the insertion locus that could impact Cre expression. To test this possibility, we aimed to identify the genomic insertions sites in these two transgenic mouse lines.

**Fig. 6:**
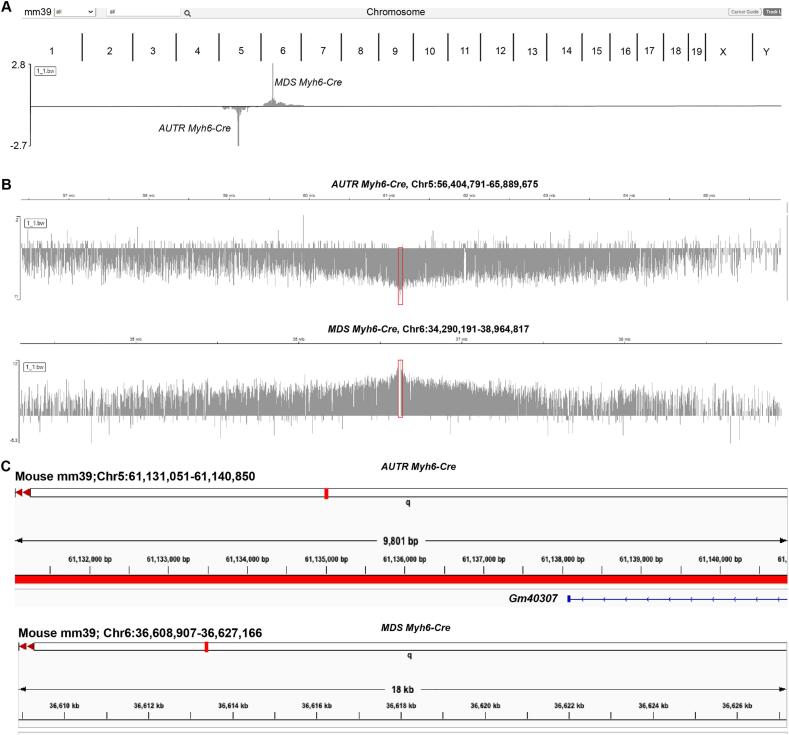
**Identification of the *AUTR Myh6-Cre* transgenic DNA insertion locus.** **A.** The chromosomes enriched with TLA signals. **B.** Zoom-in view of the *AUTR Myh6-Cre* and *MDS Myh6-Cre* TLA peaks. **C**. Genomic loci carrying the integrated transgenic DNA cassettes.

Due to the complexity of the *AUTR Myh6-Cre* transgenic construct, we failed to identify its insertion site with Thermal Asymmetric Interlaced PCR (TAIL-PCR) (TAIL) PCR [22]. We then deployed the Targeted locus amplification (TLA) method [23] to map the possible insertion locus. TLA is a technology to selectively amplify and sequence the proximal sequences around a targeted locus. After crosslinking and enzyme digestion of the genomic DNA, four primers that annealed to the Cre gene sequence were used to perform inverse PCR, and the PCR products were processed to prepare for a NGS library. After NGS, we recovered 9,201,626 mapping reads and 10,428,447 mapping reads for the *MDS Myh6-Cre* and *AUTR Myh6-Cre*, respectively. Bwa-bwasw algorithm was used to align the reads to the GRCmm39 reference, and deeptools bamcompare was used to normalize the libraries in order to identify the site of integration. Our data showed that *MDS Myh6-Cre* and *AUTR Myh6-Cre* transgenic DNA were localized on Chromosome 6 and 5 (Fig. 6A), respectively. Peaks of TLA mapping narrowed down the insertion site of *AUTR Myh6-Cre* to a 10 kb region, Chr5:61,131,051-61,140,850 (Fig. 6B), which overlaps with the partial sequence of a putative long non-coding RNA gene, GM40307 (Fig. 6C). In line with the published data [24], *MDS Myh6-Cre* was mapped to an intergenic region of chromosome 6 (chr6:36,608,907-36,627,166), where the closest predicted gene (Chrm2) is 130 kb away.

We further quantified the copy number of the *AUTR Myh6-Cre* transgenic DNA, and found that this mouse line carried four concatemerized copies of the transgenic *Myh6-Cre* (Suppl. Fig. 4), whereas the *MDS Myh6-Cre* line carries six copies of the transgenic DNA [24].

## 4 Discussion

Transgenic Cre-driver mouse lines are essential for performing tissue-specific genetic studies, and therefore it is important to know the advantages and disadvantages of the published Cre mouse lines. For heart-specific genetic studies, *MDS Myh6-Cre and AUTR Myh6-Cre* transgenic mouse lines have been widely used, both of which share the same *Myh6* promoter and differ in the transgenic integration loci. *MDS Myh6-Cre* is known for causing age-related heart failure [24], whereas this spontaneous heart defect has not been reported in *AUTR Myh6-Cre* mice, making it an attractive tool for carrying out heart-specific genetic studies. The heart-specificity of *MDS Myh6-Cre* [7] and not *AUTR Myh6-Cre* has been well-characterized with genetic reporter mouse lines, which is a valuable approach for detecting adverse recombinant events caused by transient Cre expression during development [25]. In this study, using the tdTom mouse line as a genetic reporter, we aimed to compare the tissue specificity of *MDS Myh6-Cre and AUTR Myh6-Cre* side by side. Based on our previously published data [12] and the current observations, we conclude that *MDS Myh6-Cre expresses* Cre in the heart and testis, and *AUTR Myh6-Cre* drives Cre expression in the heart, testis and other vital organs. Because all the constitutively active Myh6-Cre lines have tissue-specificity issues, if the research focuses on testing gene's function in the adult heart, inducible Myh6-Cre lines are recommended.

*Use of the tdTom reporter mouse line for comparing the tissue-specificity of two Myh6-Cre transgenic mouse lines.* As a genetic reporter, the tdTom mouse line does not express tdTom fluorescence protein until the floxed stop-cassette is removed by Cre. Once Cre activates tdTom expression in a cell, tdTom will be permanently expressed in this cell and its descendants, independent of the presence of Cre recombinase. For this reason, the tdTom mouse line has been widely used in cell fate-mapping studies to investigate how specific cell lineages behave during development and disease progression [26].

*AUTR Myh6-Cre* mouse line was published more than two decades ago, and in the original report, northern blot was performed to examine *Cre* expression in multiple adult mouse organs, including the heart, skeletal muscle, brown adipose tissue, white adipose tissue, liver and brain, showing that *Cre* mRNA was only expressed in the heart [8]. Nevertheless, in the following years, ectopic *Cre* expression of this *AUTR Myh6-Cre* transgenic mouse line has been shown in two publications: 1) at e11.5*, AUTR Myh6-Cre* primarily activated the Rosa26-LacZ reporter in the heart, and sporadically activated LacZ in the head and body trunk [27]; 2) very low levels of Cre mRNA were detected in the kidney, liver and adipose tissue of some adult male *AUTR Myh6-Cre* mice, and this occurred in the absence of any detectable Cre protein or altered expression of the floxed gene (estrogen receptor alpha-ERα) in these tissues. Moreover, Cre mRNA was not observed in tissues from adult female *AUTR Myh6-Cre* mice [28]. The original description of this line, suggests that northern blot may not be sensitive enough to detect small amounts of *Cre* mRNA, which could lead to low level recombination of susceptible alleles. However, the ER〈 study suggested that low levels of Cre mRNA expression might not lead to measurable Cre protein or altered expression of the targeted floxed allele unless Cre expression exceeds a certain threshold. Nevertheless, there remains a strong rationale for assessing Cre activity and the possibility of depletion of relevant floxed alleles in non-cardiac tissues. Our current work demonstrates that a robust genetic labelling method might be essential for fully defining the tissue-specificity of a Cre-driver mouse line.

To compare the tissue-specificity of *MDS* and *AUTR Myh6-Cre* mouse lines, we crossed them with the tdTom reporter mice and systematically examined tdTom expression in different organs. In the heart, in addition to expressing Cre recombinase in the cardiomyocytes (CM), *AUTR Myh6-Cre* also drives tdTom expression in non-CMs. The identity of the *tdTom* interstitial non-parenchymal cells is unknown, and these cells also exist in all the other examined tissues, such as liver, lung, pancreas, brown adipose tissue and spleen. Recently, a knock-in *Myh6-Cre* (*KI Myh6-Cre*) mouse line has been published, in which an IRES-Cre-wpre-polyA cassette was inserted between the translational stop codon and the 3 prime untranslated region of the endogenous *Myh6* gene [9]*.* Besides activating tdTom reporter in the cardiomyocytes, *KI Myh6-Cre* also partially triggered tdTom expression in the smooth muscle cells and displayed Cre activity in the spleen, kidney, skeletal muscle and lung [9]**.** Different from *KI Myh6-Cre* and *AUTR Myh6-Cr*e, *MDS Myh6-Cr*e did not activate the expression of reporter genes in the kidney and lung as evidenced by the published data [7] and our newly acquired ex vivo imaging data (Fig. 1). For both *KI Myh6-Cre* and *MDS Myh6-Cr*e mouse lines, no Cre activity was observed in the liver. In contrast, *AUTR Myh6-Cre* activated tdTom in the liver, with tdTom+ cells being mostly interstitial non-hepatocytes at the age of P14, and both hepatocytes and non-hepatocytes being labeled with tdTom in the adult liver. By comparing these genetic reporter data between three independent *Myh6-Cre* driver lines, we conclude that *AUTR Myh6-Cre* ectopically expresses Cre in the parenchymal and interstitial cells of multiple vital organs, and that this leaky Cre expression is not caused by the transgenic *Myh6* promoter.

Besides expressing Cre in the heart and liver, *AUTR Myh6-Cre* also expresses Cre in the brain. We examined the fluorescence signals of tdTom in different brain regions and observed that olfactory bulb, cerebral cortex, thalamus and the granular layer of the cerebellar cortex were all enriched with tdTom+ cells. Of note, *AUTR Myh6-Cre* mosaically activates tdTom expression in the brain cells. Although, we observed that *AUTR Myh6-Cre* activated tdTom in both neurons and non-neuron cells, we do not know the origin and the identity of the non-neuron cells.

The tdTom genetic reporter is a sensitive tool for assessing the ectopic Cre activity of the AUTR Myh6-Cre mouse line. Our ex vivo tdTom fluorescence studies showed that MDS Myh6-Cre;tdTom and AUTR Myh6-Cre;tdTom hearts had similar fluorescence signals, and that AUTR Myh6-Cre;tdTom liver, pancreas and brain also displayed easily detectable tdTom fluorescence. Nevertheless, these ex vivo data do not mean that AUTR Myh6-Cre ectopically expresses Cre in most of the parenchymal cells. Instead, we noticed that AUTR Myh6-Cre mosaically activated tdTom in the examined organs. Using brain as an example, we quantified the tdTom positive neurons to be approximately 20 % in the anterior cingulate cortex. In this scenario, it is impossible to detect whether the transcription products of a floxed allele would be affected by AUTR Myh6-Cre within the organs mosaically expressing Cre, unless the cells subjected to adverse recombination were purified for gene expression analysis. In line with this notion, many studies have shown that AUTR Myh6-Cre only affect the target gene expression in the heart and not in the other organs [[29], [30], [31], [32]]. In these studies, a genetic reporter was not used to test the heart-specificity of AUTR Myh6-Cre. An alternative way to scrutinize the heart-specificity of these AUTR Myh6-Cre targeted floxed alleles is genomic DNA PCR, in which a pair of primers that span the floxed region can be used for examining the integrity of the floxed allele. If the floxed allele is removed from a small cell population of an organ, this genomic DNA PCR could be sensitive enough to detect the occurrence of adverse recombinant events (Fig. 2).

*Ectopic Cre expression in AUTR Myh6-Cre may or may not cause unexpected phenotypes.* Our current data suggest that *AUTR Myh6-Cre* may drive Cre expression in a specific cell linage that could contribute to the generation of new parenchymal cells, such as hepatocytes and neurons. If the floxed allele is critical for maintaining organ homeostasis, a spontaneous cardiac unrelated phenotype may occur, and vice versa. One good example to support this hypothesis is the comparison analysis of *MDS Myh6-Cre; Yap*^*fl/fl*^ *and AUTR Myh6-Cre; Yap*^*fl/fl*^ mice, where the *MDS Myh6-Cre; Yap*^*fl/fl*^ mice survived for 2–3 months, while most of the *AUTR Myh6-Cre; Yap*^*fl/fl*^ mice mice did not survive to weaning, suggesting that the aberrant expression of *Cre* in the *AUTR Myh6-Cre*;*Yap*^*fl/fl*^ mouse line has physiological consequences. We previously reported that germline depletion of VGLL4 caused perinatal death; however *MDS Myh6-Cre* mediated depletion of VGLL4 did not cause any noticeable phenotype [33]. This observation also holds true with the *AUTR Myh6-Cre; Vgll4*^*fl/fl*^ mice, which breed and grow normally (data not shown)*,* arguing that ectopic Cre expression in the *AUTR Myh6-Cre* allele could be physiologically insignificant, as has been observed for other alleles.

Thus, whether *AUTR Myh6-Cre* causes unexpected phenotypes may depend on the nature of the floxed allele*.* This notion is not only supported by our studies of mice harboring *Yap* and *Vgll4* flox alleles but by numerous previously published studies. In many of these *AUTR Myh6-Cre* mediated knockout studies, the knockout mice only revealed a heart-specific phenotype. For example, *AUTR Myh6-Cre*-driven depletion of either Insulin receptor (INSR) [34] or IRS1/2 did not cause a systemic metabolic phenotype [35], which contrasts with significant metabolic disturbances observed following liver-specific knockout of these alleles [[36], [37], [38], [39]]. Germline Cytochrome p450 knockout mice are embryonic lethal; however, *AUTR Myh6-Cre* depletion of this gene did not cause obvious phenotype [31]. Big mitogen-activated protein kinase 1 (BMK1) is a member of the MAPK family. Germline depletion of BMK1 caused embryonic lethality between e9.5 and e10, and endothelial cell-specific ablation of BMK1 also resulted in embryonic lethality at the same developmental stage. Interestingly, both of these two strains of mutants displayed identical cardiovascular defects. In the same study, *AUTR Myh6-Cre* was used to knock out BMK1 in the cardiomyocytes, and the resulting mutants developed normally and did not show any cardiovascular defects [40]. This study demonstrated that BMK1 is an essential factor for maintaining the biology of endothelial cells, and suggests that *AUTR Myh6-Cre* does not recombine this allele in endothelial cells.

In another cohort of studies, *AUTR Myh6-Cre* derived mutants manifested both heart failure and variable survival phenotypes, rendering it challenging to determine whether the aspects of the survival phenotypes resulted from ectopic Cre expression or were secondary effects of heart failure. Unc-51-like kinase 1 (ULK1) and ULK2 are two homolog kinases regulating autophagy. *AUTR Myh6-Cre* targeted ULK1 and ULK2 double knockout mice (cU1/2-DKO) were born at a 40 % lower rate than the expected Mendelian ratio, and the basis of the perinatal loss was not determined. However surviving cU1/2-DKO mice exhibited normal cardiac function until 69-weeks of age, after which heart failure developed and premature mortality was observed [41]. Because cardiac function was not assessed in utero in this study, the basis of the perinatal loss is uncertain. Protein Kinase cAMP-Dependent Type I Regulatory Subunit Alpha (*PRKAR1A)* encodes a tumor suppressor that negatively regulates PKA activity, and *AUTR Myh6-Cre* driven loss of *Prkar1a* caused embryonic lethality*.* At e11.5, the mutant embryos were discernible by their much smaller head and body trunk size. Besides their smaller size, the mutants had major heart development defects, with the heart being dilated and myxomatous lesions formed between the thin ventricle walls [27]. Thus for certain alleles, the possibility exists that adverse phenotypes could potentially be attributable to recombination of the floxed alleles in other cell types.

### 4.1 Genomic position effect may impair the heart-specificity of AUTR Myh6-Cre mouse line

Compared with *MDS Myh6-Cre*, *AUTR Myh6-Cre* transgenic DNA carries an additional beta-actin untranslated region (UTR) downstream of the Cre gene. *AUTR* has been widely used in transgenic constructs to enhance the expression of transgenic genes [42], and it regulates the transgene expression probably through post transcriptional regulation, such as affecting mRNA stability [43] and translation efficiency [44]; however, no data are available regarding to whether *AUTR* causes the transgenic gene to express ectopically. Another difference between *MDS Myh6-Cre* and *AUTR Myh6-Cre* mouse lines is the transgenic insertion site. Here we show that *MDS Myh6-Cre* is inserted into a gene desert locus of Chromosome 6, and *AUTR Myh6-Cre* is integrated into the GM40307 gene locus on chromosome 5. Genomic position effect has been identified as an underlying mechanism contributing to aberrant transgene expression in some transgenic mouse lines [45,46]. For the *MDS Myh6-Cre* transgenic mouse line, we reason that the gene-poor intergenic locus carrying the transgenic DNA does not contain cis-regulatory elements influencing Cre expression, so that the expression of Cre is exclusively controlled by the transgenic *Myh6* promoter. In contrast, the integration site of the *AUTR Myh6-Cre* DNA construct is proximate to the gene locus of GM40307 (a putative long non-coding RNA gene), where cis-regulatory elements and epigenetic modification may affect the tissue specific expression of transgenic Cre.

### 4.2 Limitations of the current study

First, in this work, we used tdTom reporter mouse line as the tool with which to test ectopic Cre activity. Among the known fluorescent protiens, tdTom has the highest brightness, making the tdTom reporter line a very sensitive tool; however, marking cells where recombination could occur does not address the physiological significance of ectopic Cre activity. Moreover, distinct floxed alleles may respond to Cre in different ways, i.e., the genomic structure and the epigenetic context of the floxed allele could both influence recombination efficiency [47]. Therefore, although we observed aberrant Cre activity in various non-cardiac tissues of the *AUTR Myh6-Cre*;tdTom mice, we need to emphasize that leaky Cre in this *AUTR Myh6-Cre* mouse line may not necessarily be physiologically significant, which depends on the nature of the floxed allele that is being targeted. Moreover, if a subset of cells is negatively selected in organs with high regenerative capacity, such as the liver, cells which might initially not survive, could be replaced by non-Cre expressing cells. Second, our current work suggests that genomic position effect may impair the tissue-specificity of *AUTR Myh6-Cre*. At last, this study tests only the specificity of the Cre. For investigators using Cre to delete a gene in cardiomyocytes, the leaky expression of the *AUTR Myh6-Cre* may not be an issue if the gene-of-interest is only expressed in cardiomyocytes.

More work is needed to identify the responsible cis-regulatory element that could contribute to Cre ectopic expression in this mouse line and to identify the specific cell lineages that could be affected.

## Sources of funding

Z.L. was supported by 10.13039/100000050NHLBI (Grant number: 1R01HL14681001A1). N.R.T was supported by NIH R01HL170051. C.W.K. was supported by Masonic Medical Research Institute.

## CRediT authorship contribution statement

**Amanda Davenport:** Writing – review & editing, Investigation, Data curation. **Chase W. Kessinger:** Writing – review & editing, Investigation, Formal analysis, Data curation. **Ryan D. Pfeiffer:** Investigation. **Nikita Shah:** Investigation. **Richard Xu:** Investigation. **E. Dale Abel:** Writing – review & editing, Resources. **Nathan R. Tucker:** Writing – review & editing, Investigation, Formal analysis, Data curation. **Zhiqiang Lin:** Writing – original draft, Supervision, Investigation, Funding acquisition, Formal analysis, Conceptualization.

## Declaration of competing interest

The authors declare the following financial interests/personal relationships which may be considered as potential competing interests:

Zhiqiang Lin reports financial support was provided by NIH. If there are other authors, they declare that they have no known competing financial interests or personal relationships that could have appeared to influence the work reported in this paper.

## Appendix A Supplementary data

Supplementary material Image 1

## References

[1] Kim H., Kim M., Im S.-K., Fang S. (2018). Mouse Cre-LoxP system: general principles to determine tissue-specific roles of target genes. Laboratory animal research.

[2] Ray M.K., Fagan S.P., Brunicardi F.C. (2000). The Cre–loxP system: A versatile tool for targeting genes in a cell-and stage-specific manner. Cell Transplant.

[3] Mao X., Fujiwara Y., Orkin S.H. (1999). Improved reporter strain for monitoring Cre recombinase-mediated DNA excisions in mice. Proc Natl Acad Sci.

[4] Payne S., De Val S., Neal A. (2018). Endothelial-specific cre mouse models: is your cre credibile. Arterioscler Thromb Vasc Biol.

[5] Madisen L., Zwingman T.A., Sunkin S.M., Oh S.W., Zariwala H.A., Gu H. (2010). A robust and high-throughput Cre reporting and characterization system for the whole mouse brain. Nat Neurosci.

[6] Ng W.A., Grupp I.L., Subramaniam A., Robbins J. (1991). Cardiac myosin heavy chain mRNA expression and myocardial function in the mouse heart. Circ Res.

[7] Agah R., Frenkel P.A., French B.A., Michael L.H., Overbeek P.A., Schneider M.D. (1997). Gene recombination in postmitotic cells. Targeted expression of Cre recombinase provokes cardiac-restricted, site-specific rearrangement in adult ventricular muscle in vivo. J Clin Invest.

[8] Abel E.D., Kaulbach H.C., Tian R., Hopkins J.C., Duffy J., Doetschman T. (1999). Cardiac hypertrophy with preserved contractile function after selective deletion of GLUT4 from the heart. J Clin Invest.

[9] Huang X., Yan L., Kou S., Meng J., Lu Z., Lin C.P. (2021). Generation and characterization of a Myh6-driven Cre knockin mouse line. Transgenic Res.

[10] Yan J., Zhang L., Sultana N., Park D.S., Shekhar A., Bu L. (2015). A murine Myh6MerCreMer Knock-in allele specifically mediates temporal genetic deletion in cardiomyocytes after tamoxifen induction. PLoS One.

[11] Sohal D.S., Nghiem M., Crackower M.A., Witt S.A., Kimball T.R., Tymitz K.M. (2001). Temporally regulated and tissue-specific gene manipulations in the adult and embryonic heart using a tamoxifen-inducible Cre protein. Circ Res.

[12] Sheldon C., Kessinger C.W., Sun Y., Kontaridis M.I., Ma Q. (2023). Myh6 promoter-driven Cre recombinase excises floxed DNA fragments in a subset of male germline cells. J Mol Cell Cardiol.

[13] Zhang N., Bai H., David K.K., Dong J., Zheng Y., Cai J. (2010). The Merlin/NF2 tumor suppressor functions through the YAP oncoprotein to regulate tissue homeostasis in mammals. Dev Cell.

[14] Tucker N.R., Chaffin M., Fleming S.J., Hall A.W., Parsons V.A., Bedi K.C. (2020). Transcriptional and cellular diversity of the human heart. Circulation.

[15] Pan D. (2010). The hippo signaling pathway in development and cancer. Dev Cell.

[16] Del Re D.P., Yang Y., Nakano N., Cho J., Zhai P., Yamamoto T. (2013). Yes-associated protein isoform 1 (Yap1) promotes cardiomyocyte survival and growth to protect against myocardial ischemic injury. J Biol Chem.

[17] van Heukelum S., Mars R.B., Guthrie M., Buitelaar J.K., Beckmann C.F., Tiesinga P.H.E. (2020). Where is cingulate cortex? A cross-species view. Trends Neurosci.

[18] De León Reyes N.S., Bragg-Gonzalo L., Nieto M. (2020). Development and plasticity of the corpus callosum. Development.

[19] Gusel’Nikova V.V., Korzhevskiy D.E. (2015). NeuN as a neuronal nuclear antigen and neuron differentiation marker. Acta Nat.

[20] Brunjes P.C., Kay R.B., Arrivillaga J.P. (2011). The mouse olfactory peduncle. J Comp Neurol.

[21] Amaral D.G., Scharfman H.E., Lavenex P. (2007). The dentate gyrus: fundamental neuroanatomical organization (dentate gyrus for dummies). Prog Brain Res.

[22] Pillai M.M., Venkataraman G.M., Kosak S., Torok-Storb B. (2008).

[23] de Vree P.J., de Wit E., Yilmaz M., van de Heijning M., Klous P., Verstegen M.J. (2014). Targeted sequencing by proximity ligation for comprehensive variant detection and local haplotyping. Nat Biotechnol.

[24] Pugach E.K., Richmond P.A., Azofeifa J.G., Dowell R.D., Leinwand L.A. (2015). Prolonged Cre expression driven by the α-myosin heavy chain promoter can be cardiotoxic. J Mol Cell Cardiol.

[25] Song A.J., Palmiter R.D. (2018). Detecting and avoiding problems when using the Cre-lox system. Trends Genet.

[26] Kretzschmar K., Watt F.M. (2012). Lineage tracing. Cell.

[27] Yin Z., Jones G.N., Towns W.H., Zhang X., Abel E.D., Binkley P.F. (2008). Heart-specific ablation of Prkar1a causes failure of heart development and myxomagenesis. Circulation.

[28] Tham Y.K., Bernardo B.C., Claridge B., Yildiz G.S., Woon L.M.-L., Bond S. (2023). Estrogen receptor alpha deficiency in cardiomyocytes reprograms the heart-derived extracellular vesicle proteome and induces obesity in female mice. Nature Cardiovascular Research.

[29] Pereira R.O., Wende A.R., Olsen C., Soto J., Rawlings T., Zhu Y. (2014). GLUT1 deficiency in cardiomyocytes does not accelerate the transition from compensated hypertrophy to heart failure. J Mol Cell Cardiol.

[30] Noh J., Wende A.R., Olsen C.D., Kim B., Bevins J., Zhu Y. (2015). Phosphoinositide dependent protein kinase 1 is required for exercise-induced cardiac hypertrophy but not the associated mitochondrial adaptations. J Mol Cell Cardiol.

[31] Fang C., Gu J., Xie F., Behr M., Yang W., Abel E.D. (2008). Deletion of the NADPH-cytochrome P450 reductase gene in cardiomyocytes does not protect mice against doxorubicin-mediated acute cardiac toxicity. Drug Metab Dispos.

[32] Kim J., Wende A.R., Sena S., Theobald H.A., Soto J., Sloan C. (2008). Insulin-like growth factor I receptor signaling is required for exercise-induced cardiac hypertrophy. Mol Endocrinol.

[33] Sheldon C., Farley A., Ma Q., Pu W.T., Lin Z. (2022). Depletion of VGLL4 causes perinatal lethality without affecting myocardial development. Cells.

[34] Belke D.D., Betuing S., Tuttle M.J., Graveleau C., Young M.E., Pham M. (2002). Insulin signaling coordinately regulates cardiac size, metabolism, and contractile protein isoform expression. J Clin Invest.

[35] Riehle C., Wende A.R., Zhu Y., Oliveira K.J., Pereira R.O., Jaishy B.P. (2014). Insulin receptor substrates are essential for the bioenergetic and hypertrophic response of the heart to exercise training. Mol Cell Biol.

[36] Michael M.D., Kulkarni R.N., Postic C., Previs S.F., Shulman G.I., Magnuson M.A. (2000). Loss of insulin signaling in hepatocytes leads to severe insulin resistance and progressive hepatic dysfunction. Mol Cell.

[37] Dong X., Park S., Lin X., Copps K., Yi X., White M.F. (2006). Irs1 and Irs2 signaling is essential for hepatic glucose homeostasis and systemic growth. J Clin Invest.

[38] Withers D.J., Gutierrez J.S., Towery H., Burks D.J., Ren J.M., Previs S. (1998). Disruption of IRS-2 causes type 2 diabetes in mice. Nature.

[39] Kubota N., Kubota T., Itoh S., Kumagai H., Kozono H., Takamoto I. (2008). Dynamic functional relay between insulin receptor substrate 1 and 2 in hepatic insulin signaling during fasting and feeding. Cell Metab.

[40] Hayashi M., Kim S.-W., Imanaka-Yoshida K., Yoshida T., Abel E.D., Eliceiri B. (2004). Targeted deletion of BMK1/ERK5 in adult mice perturbs vascular integrity and leads to endothelial failure. J Clin Invest.

[41] Harris M.P., Zhang Q.J., Cochran C.T., Ponce J., Alexander S., Kronemberger A. (2022). Perinatal versus adult loss of ULK1 and ULK2 distinctly influences cardiac autophagy and function. Autophagy.

[42] Qin H., Gunning P. (1997). The 3′-end of the human β-actin gene enhances activity of the β-actin expression vector system: construction of improved vectors. J Biochem Biophys Methods.

[43] Lyubimova A., Bershadsky A.D., Ben-Ze’ev, A. (2000). Autoregulation of actin synthesis requires the 3’-UTR of actin mRNA and protects cells from actin overproduction. J Cell Biochem.

[44] Hüttelmaier S., Zenklusen D., Lederer M., Dictenberg J., Lorenz M., Meng X. (2005). Spatial regulation of β-actin translation by Src-dependent phosphorylation of ZBP1. Nature.

[45] Sigmund C.D. (2000). Are studies in genetically altered mice out of control. Arterioscler Thromb Vasc Biol.

[46] Guy L.-G., Kothary R., Wall L. (1997). Position effects in mice carrying a lacZ transgene in cis with the β-globin LCR can be explained by a graded model. Nucleic Acids Res.

[47] Liu J., Willet S.G., Bankaitis E.D., Xu Y., Wright C.V.E., Gu G. (2013). Non-parallel recombination limits Cre-LoxP-based reporters as precise indicators of conditional genetic manipulation. genesis.

