# Supplementary material for: Comparative analysis of two independent Myh6-Cre transgenic mouse lines
Source: J Mol Cell Cardiol Plus. Author manuscript; Available in PMC 2024 Sep 25. (PMC11423776; doi:10.1016/j.jmccpl.2024.100081)
Supplement: Supplementary file 1 [file NIHMS2021957-supplement-Supplementary_information.pdf]

Comparative analysis of two independent *Myh6-Cre* transgenic mouse lines.

Amanda Davenport[1], Chase W. Kessinger[1], Ryan D. Pfeiffer[1], Nikita Shah[1,2], Richard Xu[1], E. Dale Abel[3], Nathan R. Tucker[1], and Zhiqiang Lin[1].

1.Department of Biomedical Research and Translational Medicine, Masonic Medical Research Institute, 2150 Bleecker Street, Utica, NY, 13501;

2. College of Arts and Sciences, SUNY Polytechnic Institute, Utica, NY, 13502;

3. Department of Medicine David Geffen School of Medicine and UCLA Health.

*To whom correspondence should be addressed, email: zlin@mmri.edu

Supplemental information

Material and methods

Quantification of AUTR Myh6-Cre transgenic DNA copy number.

Liver genomic DNA extracted from wild type and *AUTR Myh6-Cre* mice were quantified and used for quantitative PCR at a concentration of 50 ng/reaction. Primers amplifying both endogenous and transgenic *Myh6* promoter: AGGGCATCCAATTTGCAGGT and ctgtgcgtgaggttaccaga. Primers amplifying mouse *Gapdh* genomic DNA: GCATCCTGACCTATGGCGTA and AGGAGCCCCATTTCCCTATCT. The Ct value of Myh6 promoter ampllicons was normalized to the Ct value of the *Gapdh* amplicons.

TLA library preparation and analysis.

Targeted Locus Amplification (TLA) to identify *Myh6-Cre* insertion sites was performed following a published protocol[1] with optimized modifications. Intact nuclei were extracted from flash frozen murine liver tissue as described[2]. 10 million nuclei were isolated from each genotype and were then immediately crosslinked with 1.0% PFA for 10 min and quenched in 300 mM glycine. After partial permeabilization in lysis buffer (50 mM Tris, 150 mM NaCl, 5 mM EDTA, 0.5% IGEPAL, 1.0% Triton X-100, pH 7.5), nuclei pellets were washed in restriction enzyme buffer for NlaIII (New England Biolabs). Nuclei were lysed by addition of 0.3% SDS and 3.0% Triton X-100 with shaking at 900 RPM for one hour at 37°C. 200U of NlaIII were added and incubated overnight at 37°C with shaking at 900 RPM. Samples were diluted, digested, and partially decrosslinked for 1 hour at 65°C using 50 µg of Proteinase K. After addition of T4 ligase buffer to 1X concentration, ligation was completed using 1,600 CEU of T4 ligase (New England Biolabs) for 2 hours at room temperature. Decrosslinking was completed with Proteinase K incubation overnight at 65°C. DNA was isolated using traditional phenol/chloroform then sodium acetate/ethanol precipitation. Secondary digest was performed using NspI in a final volume of 500 µL overnight at 37°C, with ligation performed on the resultant digest in a final reaction volume of 13 mL with 80U of T4 ligase. After sodium acetate/ethanol precipitation, the resultant ligated product was amplified for 34 cycles with Q5 polymerase (New England Biolabs) using primers specific to the Cre coding sequence. Final libraries for sequencing were fragmented and indexed using the NEBNext Ultra II FS kit (New England Biolabs) according to manufacturers recommendations. Libraries were pooled and sequenced on a single flow cell of an Illumina MiSeq 300 cycle kit at the Genomics Platform of the Broad Institute of MIT and Harvard.

While sequenced in paired end mode, each read was aligned independently using bwa-mem2 on GRCm39 mouse genome. To identify putative insertion sites, resultant alignment files were sorted and examined using samtools and bedtools genomecov. Rather than formal peak calling, the amplitude of insertion site adjacent reads compared to background is stark at each boundary of insertion, with rapid

read decrease at the site of insertion.

Cre primers utilized for TLA.

Set 1	TTACGGCGCTAAGGATGACT	TAAGCAATCCCCAGAAATGC
Set 2	GAACGTGCAAAACAGGCTCT	ATTGCTGTCACTTGGTCGTG
Set 3	GGAGTTTCAATACCGGAGAT	AGGGTGTTATAAGCAATCCC
Set 4	AGTTTCAATACCGGAGATCA	TTTCGGCTATACGTAACAGG

References:

[1] de Vree, P.J., de Wit, E., Yilmaz, M., van de Heijning, M., Klous, P., Verstegen, M.J., Wan, Y., Teunissen, H., Krijger, P.H., Geeven, G., Eijk, P.P., Sie, D., Ylstra, B., Hulsman, L.O., van Dooren, M.F., van Zutven, L.J., van den Ouweland, A., Verbeek, S., van Dijk, K.W., Cornelissen, M., Das, A.T., Berkhout, B., Sikkema-Raddatz, B., van den Berg, E., van der Vlies, P., Weening, D., den Dunnen, J.T., Matusiak, M., Lamkanfi, M., Ligtenberg, M.J., ter Brugge, P., Jonkers, J., Foekens, J.A., Martens, J.W., van der Luijt, R., van Amstel, H.K., van Min, M., Splinter, E. and de Laat, W. (2014) Targeted sequencing by proximity ligation for comprehensive variant detection and local haplotyping. *Nat Biotechnol* **32**, 1019-1025.

[2] Tucker, N.R., Chaffin, M., Fleming, S.J., Hall, A.W., Parsons, V.A., Bedi Jr, K.C., Akkad, A.-D., Herndon, C.N., Arduini, A. and Papangeli, I. (2020) Transcriptional and cellular diversity of the human heart. *Circulation* **142**, 466-482.

Supplemental Figures

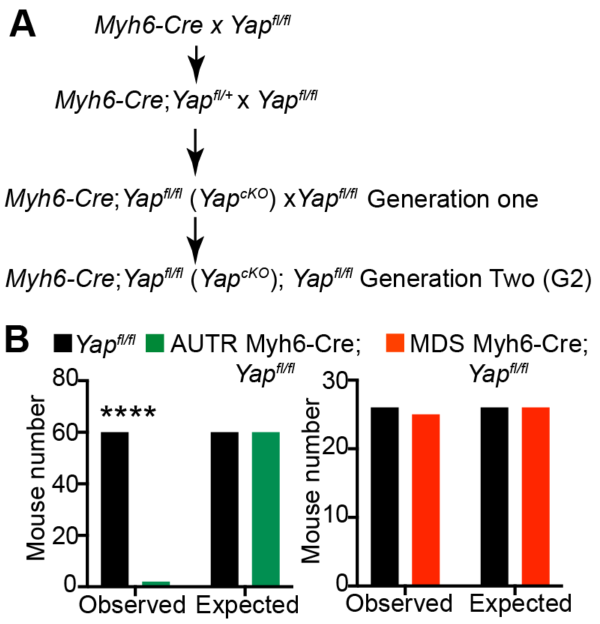

Suppl. Figure 1. Related to Figure 1.

A. Breeding strategy to acquire conditional YAP knockout mice. **B.** Comparison between observed and expected *Yap* conditional knockout mice occuring frequency in the indicated genotype. Chi-square analysis, ****, P<0.0001.

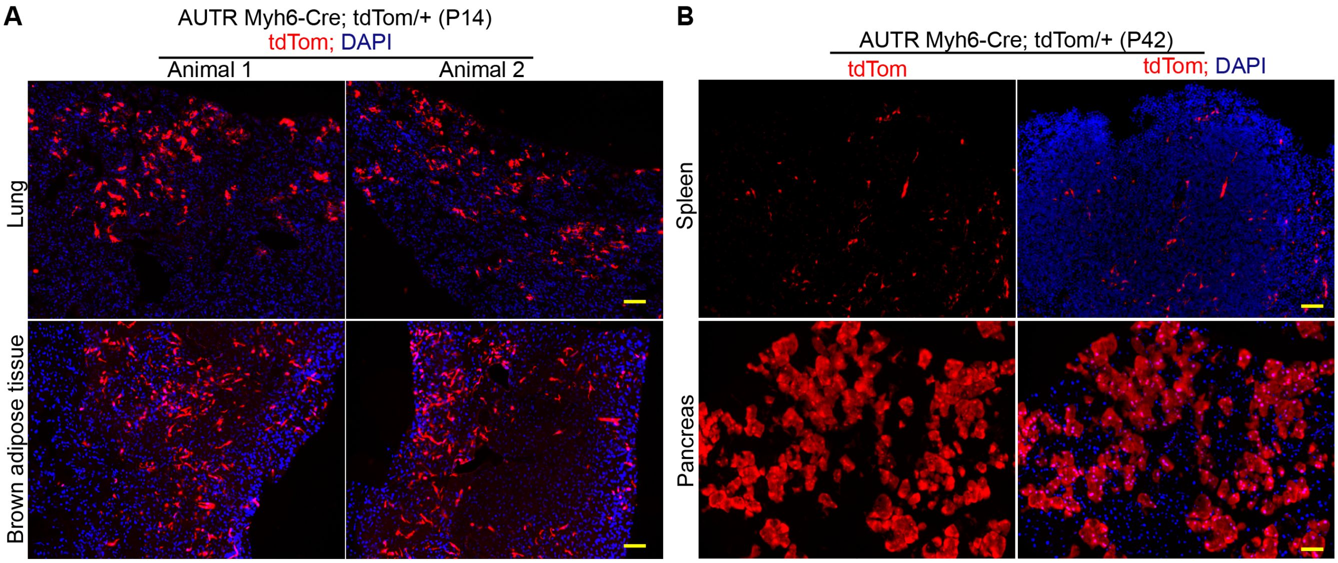

Suppl. Figure 2. Related to Figure 3. A. Fluorescence images of tissues collected from P14 mice. **B**. Fluorescence images of a spleen section**.** Spleen was collected from an adult mouse. P42: postnatal day 42. A and B, scale bar = 50 μm.

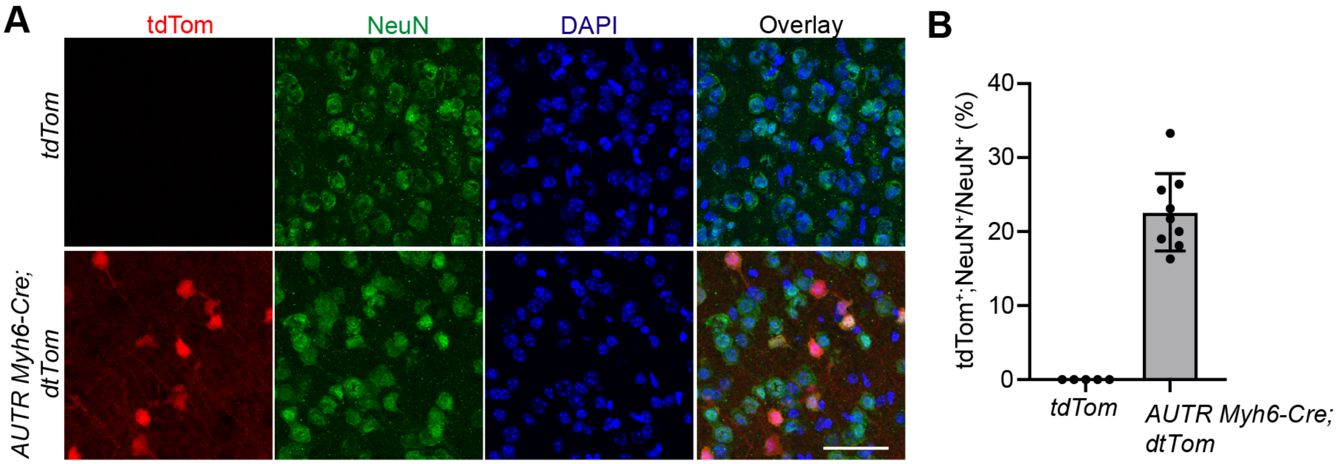

Suppl. Figure 3. Related to Figure 5. A. Immunofluorescene images of anterior cingulate cortex**.** Scale bar = 50 µm. **B.** Quantification of tdTom positive neurons. 9 images from 2 *AUTR Myh6-Cre;tdTom* mice were quantified.

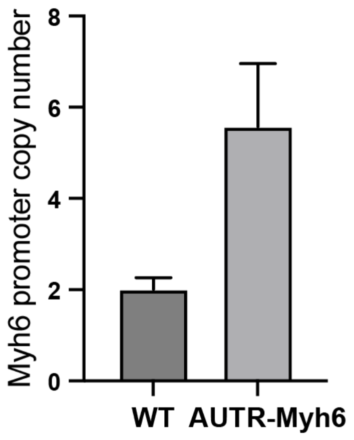

Suppl. Figure 4. Related to Figure 6. Quantification of *AUTR Myh6-Cre* transgenic DNA copy number. Liver genomic DNA was used for realtime PCR. The Ct value of *Myh6* promoter DNA amplicons was normalized to the PCR fragment of endogenous *Gapdh*. N=3 for each group.
